# Combining X-ray and neutron crystallography with spectroscopy

**DOI:** 10.1107/S2059798316016314

**Published:** 2017-02-01

**Authors:** Hanna Kwon, Oliver Smith, Emma Lloyd Raven, Peter C. E. Moody

**Affiliations:** a Henry Wellcome Laboratories for Structural Biology, Leicester Institute for Structural and Chemical Biology and Department of Molecular and Cell Biology, University of Leicester, Lancaster Road, Leicester LE1 7RH, England; b Leicester Institute for Structural and Chemical Biology and Department of Chemistry, University of Leicester, University Road, Leicester LE1 7RH, England

**Keywords:** neutron protein crystallography, single-crystal spectroscopy, photoreduction

## Abstract

X-ray protein crystallography has, through the determination of the three-dimensional structures of enzymes and their complexes, been essential to the understanding of biological chemistry. However, as X-rays are scattered by electrons, the technique has difficulty locating the presence and position of H atoms (and cannot locate H^+^ ions), knowledge of which is often crucially important for the understanding of enzyme mechanism. Furthermore, X-ray irradiation, through photoelectronic effects, will perturb the redox state in the crystal. By using single-crystal spectrophotometry, reactions taking place in the crystal can be monitored, either to trap intermediates or follow photoreduction during X-ray data collection. By using neutron crystallography, the positions of H atoms can be located, as it is the nuclei rather than the electrons that scatter neutrons, and the scattering length is not determined by the atomic number. Combining the two techniques allows much greater insight into both reaction mechanism and X-ray-induced photoreduction.

## Abstract 2

The use of neutron crystallography and *in situ* spectroscopy to study enzyme mechanism is discussed.

## 1. Introduction   

In biological processes, enzymes act as catalysts to increase the rate of reaction. These reactions always involve changes in energy and electron configurations. To understand these processes, we also need to be able to know the location of H atoms and how they are transferred between macromolecules, substrates and solvent molecules. The textbook energy profile of a reaction will show peaks of transition states (of the highest energy or least stability) and troughs of intermediates (more stable than the transition states but still less stable than reactants or products). Unfortunately, this means that the most interesting states are the least stable and the hardest to study. For crystal structures to report on mechanism we need to establish how we can isolate and monitor intermediates in the crystal; this monitoring has to be able to not only establish that a trapped intermediate is stable during the time taken for crystallographic structure determination but also that the process of data collection itself does not change it. A clear understanding of the chemistry of an enzymatic reaction also needs knowledge of the positions of H atoms. Furthermore, computational analysis and simulations of enzyme mechanisms require a full description of hydrogen positions.

## 2. Finding H atoms   

**Figure 1:**
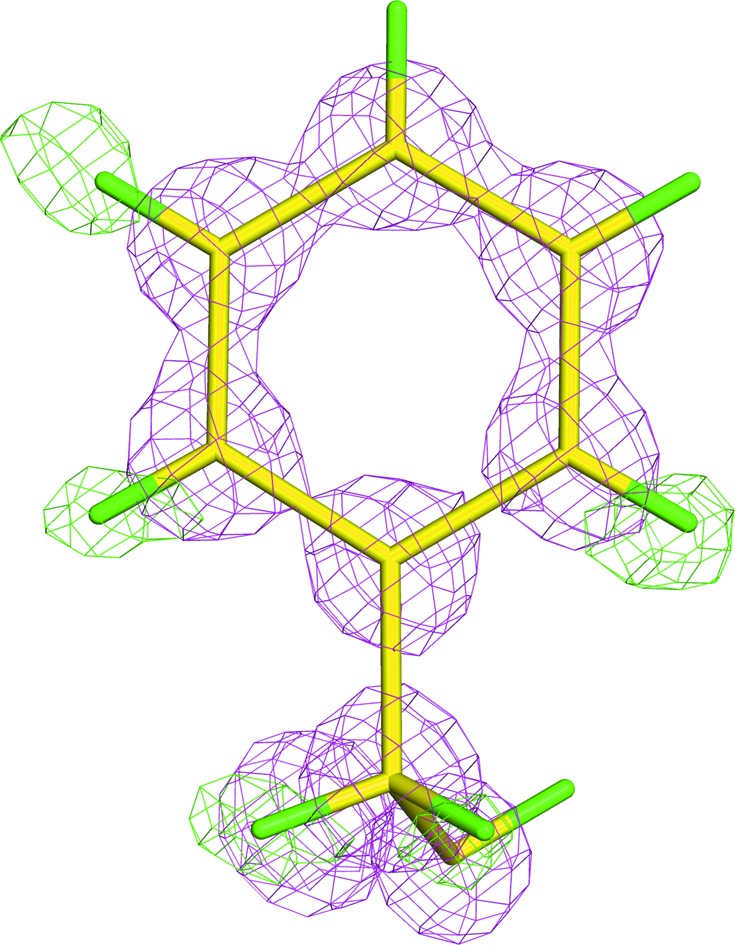
Absence of evidence is not evidence of absence. 0.9 Å resolution X-ray structure of a phenylalanine residue in PETN reductase (model and data from PDB entry 1vyr; Barna *et al.*, 2001 ▸) showing that even though some H atoms can be seen, those that are known to be present are not always observed (at one *meta* and the *para* positions in this case). The electron density is calculated excluding H atoms, the pink density is 2*F*_o_ − *F*_c_ density contoured at 3.0σ and the green density which shows both H atoms at the *ortho* position but only one at the *meta* position is *F*_o_ − *F*_c_ density contoured at 3.0σ. H atoms are shown in green.

X-ray crystallography relies on the scattering of X-rays by electrons; the scattering from an individual atom or ion is proportional to the number of electrons that it has. For crystallographic calculations the atomic electron density is described as a two-term or five-term Gaussian distribution [for further discussion, see the write-up for the *CCP*4 program *SFALL* (http://www.ccp4.ac.uk/html/sfall.html) and *International Tables for Crystallography* (2006 ▸)]. The neutral H atom has only one electron, but this will be shared when covalently bonded to a heavier atom; a positive hydrogen ion would have no electrons at all, although a discrete H^+^ ion will only have an evanescent existence. The problem of weak density is further compounded by the short length of a bond to an H atom (typically ∼1.1 Å), so resolving the very weak hydrogen electron density from a bonded ‘heavy’ (*i.e.* non-H) atom requires sub-ångstrom data. Difference maps calculated using sub-ångstrom data clearly do show density from H atoms (for a recent review of what can be seen at atomic resolution, see Neumann & Tittmann, 2014 ▸). Unfortunately, such data are not often available. When errors and the smearing effects of atomic displacement are considered, it is not safe to assume that an absence of density correlates to the absence of a hydrogen at a point (for an example, see Fig. 1 ▸). The high X-ray dose required to obtain atomic resolution data may also have an effect on the chemistry of the protein being studied (see §[Sec sec3.2]3.2).

### 2.1. Derived H-atom positions   

In the absence of atomic resolution X-ray data, some useful information may be derived by careful analysis of the chemistry and bond lengths of heavy atoms. If the electronic configuration of an atom can be confidently deduced, it may be possible to extrapolate the positions of H atoms. For example, the positions of the methylene H atoms of an *sp* ^3^ tetrahedral C atom can be extrapolated from the direction of the bonds to adjacent heavy atoms. Of course this will not work if the protonation state is unknown. In the case of a carboxylic acid group, differing bond lengths between the O atoms and the carbon would indicate protonation and distinguish C=O from C—OH. The sensitivity of difference Fourier methods gives this a power beyond what might be expected from the resolution of the data (Ahmed *et al.*, 2007 ▸).

### 2.2. Neutron crystallography   

Neutrons of suitable energy (thermal neutrons) have de Broglie wavelengths (de Broglie, 1924 ▸) that are similar to those of X-rays; thus, a beam of neutrons can be used, like X-rays, to determine the three-dimensional structures within crystals. Unlike X-rays, neutrons are scattered by the nuclei rather than the electrons. The scattering cross-sections for neutrons depend on the nuclear rather than electron density (see Table 1 ▸). A consequence of this is that deuterium, with a positive scattering length, is easily distinguished from hydrogen (with a negative scattering length) and is of similar magnitude to the other atoms in biological macromolecules. Neutron crystallography therefore allows the direct observation of hydrogen and deuterium. Obtaining neutron crystallographic data is not, however, trivial. It is very slow, especially compared with X-ray data collection at a third-generation synchrotron (with individual exposures taking hours), and it requires large crystals of the order of 1 mm^3^ and a source of thermal neutrons such as a nuclear reactor or spallation source.

The H atoms themselves also present complications. The negative scattering length of hydrogen (Table 1 ▸) is about half of the positive value for carbon, so the sum of a methylene (–CH_2_–) group will be near zero and, at medium to low resolution, this may be seen as a break in density. However, by also collecting X-ray diffraction data it is possible to refine structures simultaneously against both X-ray and neutron data. This is implemented in *PHENIX* and as a patch for *CNS* (*nCNS*; Adams *et al.*, 2009 ▸). Although the CCP4 libraries do include the nuclear scattering factors, refinement with neutron data using the *CCP*4 suite is not straightforward. H atoms also have a very large incoherent cross-section, meaning that they contribute very strongly to the background noise in the diffraction images. However, it is possible to replace the exchangeable protons by equilibrating with D_2_O and, by producing recombinant protein from a deuterated carbon source (Shu *et al.*, 2000 ▸), the non-exchangeable H atoms can also be replaced. The lack of radiation damage (see §[Sec sec3.2.3]3.2.3) by thermal neutrons means that it has been normal practice to collect data at room temperature from crystals mounted in capillaries in the manner that was used for X-ray data collection before the use of cryoconditions became standard. Although this avoids problems arising from icing over long exposure times, it is unsuitable for studying labile intermediates that require cryo-trapping.

**Figure 2:**
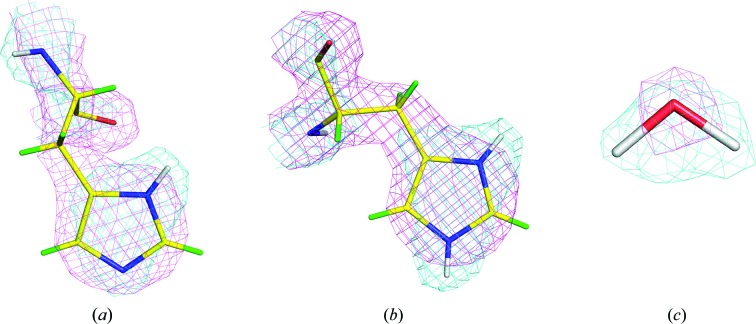
Comparison of calculated nuclear and electron densities at 2.0 Å resolution. Nuclear and electron densities are shown in cyan and magenta, respectively. (*a*) Nuclear and electron density for a tautomer of neutral histidine where N^δ1^ is deuterated. The negative scattering of the two H atoms bonded to the C^β^ of the side chain has cancelled out the nuclear density at this point. (*b*) Nuclear and electron density for a tautomer of positively charged histidine where both N^δ1^ and N^∊2^ are deuterated. (*c*) Nuclear and electron density calculated for a water molecule. All densities are contoured at 2σ. The densities were calculated in *PHENIX* (Adams *et al.*, 2009 ▸). H and D atoms are shown in green and white, respectively.

The power of neutron crystallography to show hydrogen positions and thus to understand enzyme mechanism is illustrated by the example of histidine. The side chain of this amino acid includes an imidazole ring with a p*K*_a_ near neutrality. The ability of this group to accept and donate protons is key to its involvement in enzymatic catalysis. This of course includes the famous ‘catalytic triad’ of the serine proteases (Matthews *et al.*, 1967 ▸). By H/D exchange with D_2_O the imidazole group will be either neutral and singly deuterated or positively charged and doubly deuterated. The nuclear density of these clearly shows the different ionization states (Fig. 2 ▸ *a* and 2 ▸ *b*). Water, which appears as a sphere of electron density from the O atom in X-ray data-derived maps, when substituted by D_2_O shows as a ‘banana’ shape in the neutron maps (Fig. 2 ▸ *c*). These observations allow hydrogen bonds to be assigned with confidence. For a fuller discussion of neutron protein crystallography, see Blakeley *et al.* (2015 ▸) and O’Dell *et al.* (2016 ▸).

## 3. Catching and verifying intermediates   

As the most catalytically relevant and therefore informative states of the reaction pathway of an enzyme are expected to be the least stable, ways have to be found to follow reactions in the crystal and then to manipulate conditions such that these interesting states can be trapped and maintained for structural study.

### 3.1. Single-crystal spectroscopy   

The usual method of studying enzyme reactions in solution is by electronic absorption or fluorescence spectroscopy in the UV or visible range. As a reaction proceeds and a substrate or cofactor changes its electronic state, its spectral signature changes, and in favourable cases this can be followed and analysed. The classic example is the increase in the 340 nm absorbance of NAD(P)H over NAD(P) that is used to follow dehydrogenase reactions (Krimsky & Racker, 1955 ▸).

This principle can be applied to crystals as well as solution, although there are some practical difficulties that need to be considered. The relatively small size of crystals means that the light beam needs to have an appropriately small cross-section; this is usually achieved by focusing optics. If the effects of irradiation are to be followed, it is important to have the same part of the crystal illuminated as is irradiated (a co-axial solution to this problem is described in Owen *et al.*, 2009 ▸). A crystal may act as a prism, refracting different wavelengths anisotropically and altering the spectrum as the crystal is rotated. The high concentration of protein in the crystal may result in the optical density of some features of the spectrum being very high and therefore noisy or unmeasurable (*e.g.* the Soret band in haem proteins).

A notable early achievement of the technique was to demonstrate that enzyme crystals are catalytically active, relieving anxieties that the process of crystallization somehow made them unphysiological, which would have meant that X-ray crystal structures of proteins would have been of little value. Rossi and Bernhard first used single-crystal spectroscopy to verify that the reactions of a chromophoric derivative of chymotrypsin are the same in the crystal as in solution (Rossi & Bernhard, 1970 ▸). Later, the turnover of glyceraldehyde 3-phosphate dehydrogenase in a crystal mounted in a flow cell was characterized by following the NAD/NADH 340 nm changes spectrophoto­metrically (Vas *et al.*, 1979 ▸), establishing the validity of crystal structures as representing catalytically active conformations of the protein. The standard technique of collecting data at 100 K means that once intermediates have been formed in the crystal, they can be cryo-trapped for structure determination. Combining the spectroscopic monitoring of intermediate states has allowed time-resolved determinations of the structures of enzyme catalytic intermediates (reviewed by Hajdu *et al.*, 2000 ▸). A recent example of the application of the techniques to ‘snapshot’ reaction steps in a dehydrogenase is described by Huo *et al.* (2015 ▸). Single-crystal spectroscopy facilities have been installed on synchrotron beamlines and expanded from UV–Vis absorbance to include fluorescence and Raman spectroscopy. The current status and availability of these resources are reviewed extensively in the proceedings of the 2014 CCP4 Study Weekend (Dworkowski *et al.*, 2015 ▸; von Stetten *et al.*, 2015 ▸).

### 3.2. Photoreduction   

Collecting X-ray data at 100 K may catch unstable intermediates for study, but it does not prevent the primary photoreduction events caused by ionizing radiation during data collection, nor does the low temperature prevent the movement of electrons. Radiation-induced damage in protein crystals has been described and reviewed extensively by Garman (2010 ▸). The chemical activity of the catalytic processes in an enzyme means that these are especially vulnerable to perturbation. X-ray photons have energies in the range 6–20 keV, about 1000 times the electron first ionization energy of most atoms (between 4 and 16 eV). The X-ray photons will cause the ejection of electrons from atoms (Einstein, 1905 ▸; Meitner, 1922 ▸). Ejected electrons may travel considerable distances within the protein structure and are attracted to electrophilic centres in redox enzymes. There are many examples of structures in the PDB that are described as having a particular oxidation state but have most probably been reduced to a greater or lesser extent during data collection. If the spectrum from the crystal (or irradiated portion of the crystal) is monitored during data collection, it becomes possible to be confident of the redox state of the structure being determined.

#### 3.2.1. The example of flavoenzymes   

**Figure 3:**
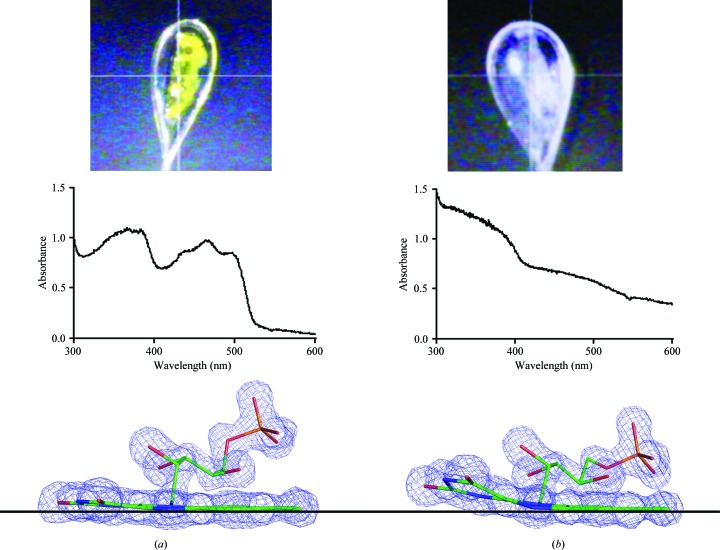
The effects of photoreduction in PETN reductase. (*a*) A crystal in the oxidized form, the spectrum and the near-planar electron density of the isoalloxazine ring of the FMN molecule. (*b*) A crystal that has been reduced by X-ray data collection, the resulting spectrum and the bent isoalloxazine ring of the FMN. 2*F*_o_ − *F*_c_ electron density contoured at 2σ is shown as a blue mesh. Crystallization conditions are shown in Table 2 ▸, data-collection statistics are shown in Table 3 ▸ and refinement statistics are shown in Table 4 ▸.

The flavoenzymes provide an interesting example whereby very few deposited structures have had their redox states verified during or after data collection. The ‘butterfly bend’ of the flavin (the dihedral angle between the planes formed by the pyrimidine and benzene rings of the isoalloxazine moiety) depends on the oxidation state, with the oxidized form being flat and reduction inducing a bend along the N5–N10 axis (Fig. 3 ▸). Any discussion of enzyme mechanism deduced from the structure clearly needs to be in the context of the oxidation state observed. The photoreduction of the flavin in an FMN-containing enzyme during X-ray data collection was followed by Røhr *et al.* (2010 ▸), showing the flavin bend. We also observed this effect in crystals of PETN reductase; Fig. 3 ▸ shows the difference between the yellow colour of the oxidized enzyme and the colourless X-ray reduced form, and the spectra from the two crystal states. By monitoring the crystal spectrum after varying X-rays doses we were able to use a multi-crystal strategy to obtain a minimally reduced structure and compare this with the X-ray reduced structure, showing clearly that caution is required when interpreting structures that have not been validated with spectroscopy. Many of the structures of flavoenzymes in the PDB appear to have used the restraints for the oxidized flavins (FAD and FMN) in the refinement, even when the authors believe they have the reduced form (and so should use the FDA or FNR monomer library entries). The limited and inconsistent descriptions within the CCP4 monomer libraries (http://www.ccp4.ac.uk/html/lib_list.html) may add to the confusion.

#### 3.2.2. The example of haem peroxidases   

The haem peroxidase reaction has two intermediate states (compound I and compound II) where the haem iron is in the highly oxidized ferryl (Fe^IV^) state (Kwon *et al.*, 2016 ▸). In order to fully understand the reaction, it is necessary to determine whether these intermediates are protonated, *i.e.* Fe^IV^=O or Fe^IV^—OH. It is possible to monitor the formation of the intermediates spectroscopically and show that these are sufficiently stable to be cryo-trapped (Gumiero *et al.*, 2011 ▸). Furthermore, using single-crystal spectrophotometry it can be shown that this can be performed in the crystal. Berglund *et al.* (2002 ▸) determined the structures of horseradish peroxidase intermediates and showed the changes in redox states during X-ray data collection. This was performed by careful monitoring and merging together of data from several crystals with differing levels of exposure. In the case of cytochrome *c* peroxidase, early work on the compound I intermediate reported varying Fe—O distances of 1.7–1.9 Å (Fülöp *et al.*, 1994 ▸) and 1.87 Å, suggesting Fe—OH (Bonagura *et al.*, 2003 ▸). However, subsequent work using the low-dose multi-crystal approach showed that the distance was 1.63 Å (Gumiero *et al.*, 2011 ▸), consistent with Fe=O. Comparing the structures derived from crystals with increasing doses showed a steadily increasing distance to ∼2.0 Å (Gumiero, 2011 ▸). The use of distance to extrapolate bond order and therefore protonation state has to be treated with caution. Incomplete, low-resolution or poor-quality data, as well as incomplete models or high *B* values, will increase the uncertainties in atomic positions (Murshudov & Dodson, 1997 ▸). A reasonable estimate of this positional uncertainty (ESU) requires the data to be of sufficient resolution to be able to calculate a full matrix inversion in *SHELX* (Sheldrick, 2008 ▸). Furthermore, in the case of adjacent atoms with very different electron densities [*e.g.* iron (*Z* = 26) and oxygen (*Z* = 8) in this case] series-termination errors in the Fourier transform may introduce significant shifts in atomic positions (Fülöp *et al.*, 2000 ▸).

#### 3.2.3. Neutron crystallography of haem peroxidases   

Neutron crystallography has the advantage of being able to directly observe H or D atoms and does not induce photoreduction. The haem-containing oxygen-binding protein myoglobin was one of the earliest to be studied by neutron crystallography, showing for the first time the iron–histidine hydrogen bond directly (Phillips & Schoenborn, 1981 ▸). Recently, neutron crystallography has been used to resolve the catalytic mechanism of the haem peroxidases by the direct observation of H atoms. By reacting crystals (as before) that have been soaked in D_2_O to exchange H/D and verifying the intermediate formation and stability at 100 K with single-crystal spectroscopy and EPR, neutron crystallography could be undertaken on compound I and compared with ferric cytochrome *c* peroxidase. Owing to the moderate resolution of the structures and as only the exchangeable H atoms were replaced by deuterium, it was necessary to include X-ray data in the refinement using *PHENIX* (Adams *et al.*, 2009 ▸) to position the methylene groups. Neutron crystallography clearly showed that ferryl O atom was unprotonated, and showed the ionization states of the histidine residues (Casadei *et al.*, 2014 ▸). The unexpected observation that the distal histidine was doubly deuterated meant that the mechanism has had to be re-evaluated (Groves & Boaz, 2014 ▸).

#### 3.2.4. Neutron crystallography in other enzymes   

Although the cytochrome *c* peroxidase example above is the first use of the combination of cryo-trapping intermediates and neutron crystallography, neutron crystallography has, by placing H atoms, provided insight into other enzyme mechanisms. Recent examples include showing the movement of hydrogen in the reaction of xylose isomerase (Kovalevsky *et al.*, 2010 ▸), the protonation state of the bound substrate in urate oxidase (Oksanen *et al.*, 2014 ▸) and the details of the interactions of a clinical inhibitor of HIV-1 protease (Weber *et al.*, 2013 ▸). A notable early example is the demonstration of the doubly protonated state of the catalytic histidine residue (and therefore its role as a base in the hydrolysis reaction) in the serine protease trypsin, resolving a longstanding controversy about the role of the Asp–His pair of the catalytic triad (Kossiakoff & Spencer, 1980 ▸, 1981 ▸). Bau (2005 ▸) reviews the insight gained from this and other examples.

## 4. Concluding remarks   

Single-crystal spectroscopy can allow the monitoring and validation of intermediate catalytic states in the crystal. In favourable circumstances these can be cryo-trapped and the structures determined. Single-crystal spectroscopy is also useful to monitor crystals for photoreduction during X-ray data collection. X-ray crystallography does not directly give the positions of H atoms, but neutron crystallography does. Neutron crystallography also does not induce photoreduction. However, neutron crystallography is not as accessible as X-ray crystallography. A carefully considered combination of methods is likely to be the best approach to understanding biological chemistry through structure, and new techniques (such as X-ray free-electron lasers) will increase the scope of what can be discovered.

## Supplementary Material

PDB reference: PETN reductase, oxidized, 5lgx

PDB reference: reduced, 5lgz

## Figures and Tables

**Table 1:** Atomic number (*Z*) and nuclear scattering lengths of common atoms found in biological structures Values are taken from Myles (2006 ▸).

Atom	*Z*	Incoherent cross-section (barns)	Coherent scattering length (×10^−12^ cm)
^1^H	1	80.2	−0.374
^2^D	1	2.05	0.667
^12^C	6	0.00	0.665
^14^N	7	0.49	0.937
^16^O	8	0.00	0.580
^32^S	16	0.00	0.28
^56^Fe	26	0.00	1.012

**Table 2:** Crystallization conditions for PETN reductase

Method	Vapour diffusion
Plate type	Sitting drop
Temperature (K)	298
Protein concentration (mg ml^−1^)	10
Buffer composition of protein solution	50 m*M* potassium phosphate pH 7.0
Composition of reservoir solution	25% PEG 3000, 0.1 *M* sodium citrate, 0.1 *M* cacodylate pH 6.2, 17% 2-propanol
Volume and ratio of drop	4 µl, 1:1
Volume of reservoir (µl)	500

**Table 3:** Data-collection and processing statistics for oxidized and reduced PETN reductase Values in parentheses are for the outer shell.

	Oxidized (PDB entry 5lgx)	Reduced (PDB entry 5lgz)
Diffraction source	Rotating anode	Rotating anode
Wavelength (Å)	1.5418	1.5418
Temperature (K)	100	100
Crystal-to-detector distance (mm)	40	40
Rotation range per image (°)	0.5	0.5
Total rotation range (°)	First 60° per crystal × 3	180
Exposure time per image (s)	30	30
Space group	*P*2_1_2_1_2_1_	*P*2_1_2_1_2_1_
*a*, *b*, *c* (Å)	56.69, 68.64, 88.60	56.85, 68.65, 88.93
α, β, γ (°)	90.0, 90.0, 90.0	90.0, 90.0, 90.0
Resolution range (Å)	20.82–1.49 (1.52–1.49)	21.51–1.50 (1.53–1.50)
Total No. of reflections	252795	223446
No. of unique reflections	53305	54598
Completeness (%)	94.3 (76.5)	96.8 (88.1)
Multiplicity	4.7 (3.0)	4.1 (3.0)
〈*I*/σ(*I*)〉	10.1 (2.3)	11.7 (3.0)
*R*_meas_	0.15 (0.61)	0.08 (0.44)
Overall *B* factor from Wilson plot (Å^2^)	5.6	4.5

**Table 4:** Structure solution and refinement for oxidized and reduced PETN reductase Values in parentheses are for the outer shell.

	Oxidized (PDB entry 5lgx)	Reduced (PDB entry 5lgz)
Resolution range (Å)	20.82–1.49 (1.53–1.49)	12.49–1.50 (1.52–1.50)
Completeness (%)	94.0 (79.2)	96.1 (85.0)
σ Cutoff	None	None
No. of reflections		
Working set	50507 (3118)	54130 (2241)
Test set	2715 (163)	2748 (127)
Final *R*_cryst_	0.16 (0.26)	0.14 (0.18)
Final *R*_free_	0.20 (0.28)	0.18 (0.21)
No. of non-H atoms		
Protein	2927	2976
Ligand	47	39
Water	618	673
Total	3592	3688
R.m.s. deviations		
Bonds (Å)	0.014	0.016
Angles (°)	1.41	1.36
Average *B* factors (Å^2^)		
Protein	9.21	10.9
Ligand	25.7	7.5
Water	24.0	26.5
Ramachandran plot		
Most favoured (%)	97	96
Allowed (%)	3	4
